# Personalized Office Lighting for Circadian Health and Improved Sleep

**DOI:** 10.3390/s20164569

**Published:** 2020-08-14

**Authors:** Charikleia Papatsimpa, Jean-Paul Linnartz

**Affiliations:** 1 Electrical Engineering Department, Eindhoven University of Technology, 5600 MB Eindhoven, The Netherlands; 2 Signify, 5656 AE Eindhoven, The Netherlands; j.p.linnartz@tue.nl

**Keywords:** circadian light, circadian clock, smart buildings, office, sleep

## Abstract

In modern society, the average person spends more than 90% of their time indoors. However, despite the growing scientific understanding of the impact of light on biological mechanisms, the existing light in the built environment is designed predominantly to meet visual performance requirements only. Lighting can also be exploited as a means to improve occupant health and well-being through the circadian functions that regulate sleep, mood, and alertness. The benefits of well-lit spaces map across other regularly occupied building types, such as residences and schools, as well as patient rooms in healthcare and assisted-living facilities. Presently, Human Centric Lighting is being offered based on generic insights on population average experiences. In this paper, we suggest a personalized bio-adaptive office lighting system, controlled to emit a lighting recipe tailored to the individual employee. We introduce a new mathematical optimization for lighting schedules that align the 24-h circadian cycle. Our algorithm estimates and optimizes parameters in experimentally validated models of the human circadian pacemaker. Moreover, it constrains deviations from the light levels desired and needed to perform daily activities. We further translate these into general principles for circadian lighting. We use experimentally validated models of the human circadian pacemaker to introduce a new algorithm to mathematically optimize lighting schedules to achieve circadian alignment to the 24-h cycle, with constrained deviations from the light levels desired for daily activities. Our suggested optimization algorithm was able to translate our findings into general principles for circadian lighting. In particular, our simulation results reveal: (1) how energy constrains drive the shape of optimal lighting profiles by dimming the light levels in the time window that light is less biologically effective; (2) how inter-individual variations in the characteristic internal duration of the day shift the timing of optimal lighting exposure; (3) how user habits and, in particular, late-evening light exposure result in differentiation in late afternoon office lighting.

## 1. Introduction 

Light is known to influence human physiology and behavior, as it directly stimulates the human biological clock. This endogenous circadian (∼24 h) rhythm generates the daily rhythms of rest and activity and is responsible for modulating the daily rhythms in several physiological phenomena, including the sleep/wake cycle, hormone secretion, and subjective alertness and performance in humans [1]. The timing of sleep is not only regulated by circadian phenomena, but induced by a combination of circadian and homeostatic mechanisms—in other words, sleep onset results from the combination of the circadian timing and the physiological need for sleep [2]. For millennia, our daily habits and routines were determined by the sun. We have been using the daily pattern of light and dark to set the timing of the biological clock in synchrony with the 24 h Earth rotation [3]. However, this pattern is disrupted by the modern lifestyle. Currently, the average person spends most of their time indoors (on average, 87% [4]) with a large part of the day spent at work. During the work day, the light levels indoors in offices are commonly much lower than outdoors, while evening light levels, not only from artificial illumination sources but also from smartphones or TVs, are relatively high. Additionally, most people typically delay their bedtimes and use an alarm to comply with work schedules and other social constraints. This modern lifestyle, particularly its socially enforced shifts in sleep schedules, is often seen as a cause of the mismatch with our endogenous circadian rhythmicity. In fact, the majority the world’s population is estimated to have social jet-lag [5]—that is, a circadian rhythm that is out of phase with people’s daily schedules. Social jet-lag indirectly disturbs the natural cycle of our biological clock and can have a profound effect on our mental and physical health. In fact, there are more than 100 studies that relate circadian disruption to a wide variety of health risks and diseases, including mood disorders, depression, diabetes, obesity, cardiovascular disease, and cancer [6,7,8,9,10]. Despite this growing scientific understanding of the impact of light on biological mechanisms, supported by field studies on how light induces vitality and alertness even during office hours [11,12], the benefits of this understanding are not (yet) harvested by practical systems. Office lighting is presently designed only to meet visual requirements, providing the amount of light that is “suitable and sufficient” to perform certain tasks and reduce visual discomfort. Less attention has been given to the biological effects of light, especially how it could be used to promote occupants’ health and well-being through the circadian functions that regulate sleep, mood, and alertness. Artificial light can be exploited as a means to modulate sleep-wake schedules and re-align the internal clock with the environment by phase-shifting the biological clock. Light is often mentioned to be the main synchronizer of the human biological clock. In fact, depending on the timing and magnitude of light exposure, light either accelerates (advances) or slows down (delays) the phase of the clock. As a result, a lighting system that can shift our body’s internal perception of time can potentially enhance wellbeing and can mitigate sleep problems in an attractive, unobtrusive way. In fact, a recent study in office buildings [13] showed that office workers who received high circadian stimulation in the morning reported better sleep and fewer depressive symptoms than those who received low circadian stimulation in the morning. While the importance of reducing evening light exposures to maintain a regular sleep–wake cycle is already widely known, this study also demonstrated the benefit of providing circadian stimulation throughout the entire workday. 

This paper is an extension of work originally presented in the 16th International Conference on Intelligent Environments [14]. We suggest a smart lighting control system centered towards bringing support to employees’ biological rhythm. This requires not only insights into how humans experience light but also demands optimization algorithms based on quantified and scientifically proven models that are executed by automated lighting control systems. We exploit one of the most widely adopted models of the biological clock—namely, the Jewett–Forger–Kronauer (JFK) model [15]—to introduce a novel optimization algorithm that finds the best office lighting profile to re-align the body’s natural rhythm to the 24-h cycle. This requires us to align the socially induced (by working times) sleep-wake cycle to the natural circadian sleep-wake cycle. Strategically timed light schedules are able to introduce shifts in the circadian mechanism (advance or delay the clock), which in turn enables one to synchronize the body’s natural rhythm to one’s social schedule. We can significantly enhance lighting systems by better understanding, modelling, and smartly exploiting the health benefits of light and its impact on human physiology and mental wellbeing. In fact, one can simultaneously address energy-conservation goals, improve comfort, avoid fatigue, and support task performance. In particular, if it also takes circadian effects into account, Human Centric Lighting may safeguard the health and wellbeing of employees, and it can reduce the long-term health problems associated with circadian disruption, including sleep problems, mood disorders, diabetes, obesity, cardiovascular disease, and cancer [13]. The main contributions of this paper are:

- We describe a robust mathematic method which exploits experimentally validated mathematical models of the biological clock and sleep regulation—namely, the Jewett–Forger–Kronauer and Phillips Robinson models—to derive optimal light schedules during office hours.
- We formulate the lighting control system as a mathematical optimization problem which is highly nonlinear. To this end, we introduce an iterative numerical optimization algorithm to achieve a solution.
- We show how energy constrains and user preferences drive the shape of the optimal lighting profiles by dimming the light levels during the time window when light is less biologically effective.
- We test the inter-person variability in the optimum light exposure, and conclude that the theory predicts that there is no one-size-fits-all light recipe. In particular, individual differences in the late-evening light exposure and sleep chronotype require differentiation in the late afternoon. In fact, we believe that this explains the experimental findings that generic solutions are less effective than hoped for, and this paves the way to more effective Human Centric Lighting that centers on the individual rather than on a fictitious, population-average person.

## 2. Biological Clock and Sleep

In humans, sleep is regulated by two interacting, coupled mechanisms [16]: (1) the biological clock, which generates a circadian rhythm in sleep-wake propensity, which we refer to as the circadian mechanism C; (2) a homeostatic process, H, that represents the sleep dept which builds up during wakefulness and dissipates during sleep. The circadian factor is evident by the clock-regulated timing of physiological processes—for example, the secretion of hormones like melatonin or the body temperature rhythm—while the homeostatic component is evident in sleep deprivation—i.e., the rising need for sleep after a sustained period of wakefulness. Light affects sleep through the circadian component. Light exposure throughout the day determines the circadian phase relative to the local clock time, which in turn influences the timing of key physiological properties, like the nadir of the core body temperature or the start of melatonin secretion. On the other hand, the timing of sleep influences the light input signal to the pacemaker, as, during sleep, no light reaches the eye, while during wakefulness, the amount of light that reaches the eye depends on the clock time.

### 2.1. Mathematical Model of The Human Circadian Pacemaker 

To model the circadian mechanism, we adopt the most widely accepted model of the circadian pacemaker, namely the Jewett–Forger–Kronauer model [15], as this has been extensively validated by both controlled laboratory [17,18,19] and daily living conditions studies [20,21]. The model describes the mechanisms by which light trains the self-sustaining circadian rhythmicity. Here, the circadian process is modeled as a limit-cycle oscillator that, in the absence of external light stimuli, oscillates with an intrinsic period that is close but not exactly equal to 24 h. It is only through the presence of light stimuli that the oscillator entrains to the 24 h light and dark cycle. Due to genetic variance—i.e., variations in the intrinsic circadian period—people may synchronize in a different way to the same light-dark pattern earlier or later. Mathematically, the oscillation is described by a pair of interacting state variables (*x*, *y*), described by the continuous differential equations:

(1) $$\dot{x}=\frac{\pi }{12}\left[y+\mu \left(\frac{1}{3}x+\frac{4}{3}x^{3}-\frac{256}{105}x^{7}\right)+B\right],$$

(2) $$\dot{y}=\frac{\pi }{12}\left\{qBy-\left[{\left(\frac{24}{\tau_{x}0.99729}\right)}^{2}+kB\right]x\right\},$$

where:

(3) B=Gα(1−n)(1−bx)(1−by).

The intrinsic (endogenous) period of the oscillator is defined as τ (24.18 h on average), the stiffness (dampening) of the oscillator as μ, and n is the fraction of activated photoreceptors. Inter-individual differences in the intrinsic circadian period lead to differences in the established phase of entrainment. If the circadian period is slightly shorter than 24 h—say, 23.8—then the period of the biological clock has to be delayed or lengthened by 12 min to match the 24 h Earth rotation, while if the circadian period is longer it has to be advanced or shortened. In fact, in this paper we show that differences in the circadian period lead to different sensitivity to changes, either intentional as light interventions or by social activities, which are so different that different light recipes are needed.

The effect of light is incorporated into the model through the light drive term B to describe how the light intensity I observed in the retina causes changes in the circadian parameters (speed and/or amplitude). The effect of light on the speed and amplitude depends on the (internal) timing of light exposure. When light is perceived during the late subjective night, the pacemaker is accelerated, resulting in a phase-advance, whereas light received during the early subjective night delays the pacemaker. A light and dark adaptation mechanism is incorporated in the model to describe the physiological process by which light initiates a chemical reaction within the photo-pigments of the retinal photoreceptors. This process can be thought of as comprising a pool of photoreceptors that can be either in the used (n) or ready (1−n) state. The photoreceptors are activated by light at a rate α, given by the updated model described in [20], and deactivated with a constant rate β.

(4) $$\alpha =a_{0}\sqrt{\frac{I}{9500}}\frac{I}{I+100},$$

(5) $$\dot{n}=60\left[\alpha \left(1-n\right)-\beta n\right],$$

where the values of $a_{0}$, β, and G have been determined from the experimental data. 

### 2.2. Model of the Sleep Mechanism

The second oscillator describes the physiological need for sleep, known as the homeostatic sleep pressure, which builds up during wakefulness and dissipates during sleep. We adopt the Phillips–Robinson model [22], as it has been extensively tested against human data. Specifically, sleep and wake states occur as a result of mutual interaction between sleep promoting and wake promoting neurons, as described by equations for their mean electric potential, $V_{v}$ and $V_{m}$, respectively:

(6) $$\tau_{v}\dot{V_{v}}+V_{v}=-v_{vm}\;Q_{m}+D_{v},$$

and

(7) $$\tau_{m}\dot{V_{m}}+V_{m}=-v_{mv}\;Q_{v}+D_{m},$$

where

(8) $$Q_{j}=\frac{Q_{max}}{1+\exp\left(\frac{\vartheta -V_{j}}{\sigma }\right)}.$$

This describes the relationship between the potential and the firing rate of the neurons, j = v, m, where m stands for wake-promoting and v for sleep-promoting neurons, respectively. Here, $Q_{max}=100$ is the maximum possible firing rate, ϑ=10 is the mean firing threshold, and σ=3 is the standard deviation of ϑ. The parameters $\tau_{v,m}$ are the time constants of the neuronal process and the parameters $v_{vm,\;mv}$ weight the input from population m to v and v to m, respectively. The homeostatic process is directly linked to high sleep-promoting neuron activity and is governed by the sleep firing rate and the parameter $\mu_{H},$ which measures the rate of homeostatic rise during wakefulness.

(9) $$\chi \dot{H}+H=\mu_{H}Q_{v}.$$

The homeostatic dampening factor $\mu_{H}$ and circadian clock sensitivity $v_{vc}$ are age-related parameters that account for changes in sleep timing and duration across the lifespan, but may be considered constant given the age. The parameter χ is the time constant of the process. The drive of the sleep-promoting neurons, $D_{v}$, consists of both homeostatic and circadian components—namely:

(10) $$D_{v}=v_{vc}C+v_{vh}H+D_{0},$$

where H is the homeostatic sleep pressure modelled as the level of some somnogenic chemical such as adenosine, and C represents the circadian factor. The parameter $v_{vh}$ is a constant which measures the sensitivity to the homeostatic process, $v_{vc}\;$is the sensitivity to the circadian process, and $D_{0}$ is a constant offset. Finally, wake-up and sleep onset events are represented by the Heaviside step function, which means that a person wakes up if the firing rate of wake-promoting neurons, $Q_{m}$, is greater than a threshold value $Q_{th}=1\;s^{-1}$.

(11) $$S=H\left(Q_{m}-Q_{th}\right)=\{\begin{array}{c} 1\;\left(awake\right),\;\mathrm{if}\;Q_{m}\geq Q_{th}\\ 0\;\left(sleeping\right),\;\mathrm{otherwise} \end{array}.$$

Consequently, spontaneous wake-up events are defined as the times ($t_{w\_sp}$) that 0-to-1 transitions occur, while sleep onset events are defined as the times ($t_{s\_sp}$) that 1-to-0 transitions occur.

(12) $$t_{w\_sp}=t_{S_{0-to-1}},\;t_{s\_sp}=t_{S_{1-to-0}}.$$

In coupling the two models—i.e., the circadian rhythm model described by Equations (1)–(5) to the sleep/wake model described by Equations (6)–(12), the circadian process C is assumed to be approximately sinusoidal. The original model, developed by modelling light intensity as sinusoidal, assumes C=(1+y)/2. However, using realistic light profiles for the light intensity input, Skeldon et al. [23] (suppl. mat) found that a phase shift was required to reproduce typical observed values for sleep duration and timing. We use a phase-shifted version of C to match our experimental acquired light data and sleep-wake timings, as described in [24]—namely:

(13) C=0.5(1+0.8x−0.55y).

**Figure 1:**
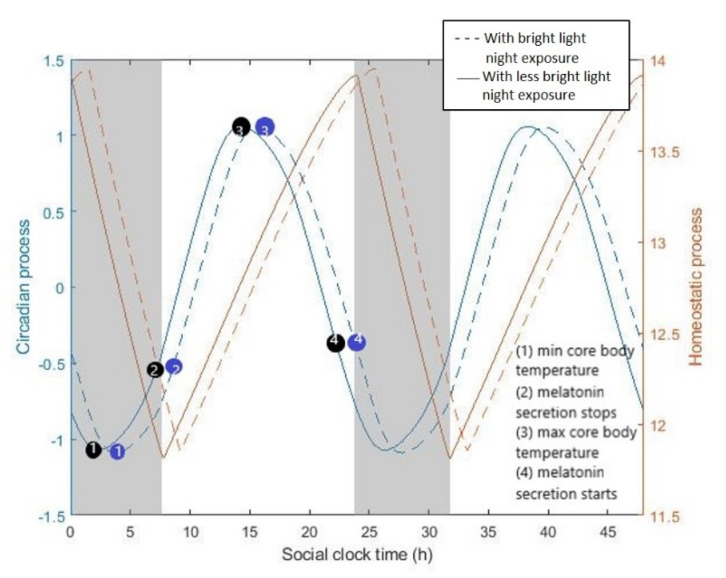
Schematic of the typical oscillation profile of the two processes that regulate sleep. The homeostatic sleep pressure H increases during wakefulness and declines during sleep, while the circadian process C describes the regulation of the body’s internal biological processes and alertness levels. The natural sleep-wake cycle (shaded region) is indicated by the interaction of homeostatic and circadian components, as described by Equation (10). The lighting profiles used as input to the model are presented in Figure 2a (far from window and bright light night exposure and far from window and no bright light night exposure, respectively).

All the model parameters are listed in Table 1. Figure 1 shows a typical oscillation profile for the circadian rhythm and the homeostatic sleep pressure. The circadian process *C* is the regulation of the body’s internal biological processes and alertness levels, and the homeostatic sleep pressure H causes a pressure to fall asleep. The natural sleep-wake cycle is indicated by the interaction of the homeostatic and circadian process, as described by Equation (10). Exposure to artificial light during the night delays the circadian process, thus the timing of key physiological properties—such as the nadir of core body temperature or the start of melatonin secretion—happen out of phase with sleep timing (grey shaded area).

### 2.3. Effect of Light on Circadian Rhythm and Sleep 

**Figure 2:**
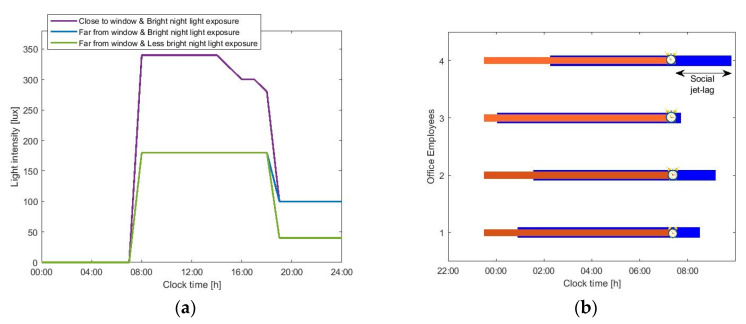
(**a**) Lighting levels representative of typical lighting exposure in the modern era. Light intensity levels of typical activities (watching TV, laptop use) were measured using a BH1750 Digital Light Sensor with an artificial light on (average value 100 lux) and off (average value 40 lux), respectively; (**b**) experienced social jet-lag in modern society, quantified as the time difference between the unconstrained sleep and wake-up times (blue bars) and sleeping and waking up under the constrains of working times (orange bar).

Here, we use the mathematical models to explore the effect of light in the modern era on circadian rhythms and sleep. To do so, we use typical indoors lighting levels (Figure 2a) to compare the natural wake-up times to the typically reported wake-up and sleep times of office employees. We consider the spontaneous (un-constrained) wake-up times estimated by the model as indicative of (or close to) a person’s entrainment phase. In that case, the difference between the unconstrained wake-up times and waking up under the constrains of working times (using an alarm) is considered as a good approximation of the amount of strain that social constrains apply on the circadian system. Our results in Figure 2b suggest that, in the absence of social constraints, we would sleep and wake up later to remain aligned with our delayed biological clock (orange bars compared to the blue ones). A wake-up effort is required if waking (enforced by the alarm) occurs far from the spontaneous wake time. Here, inter-individual differences are significant. People that adhere to recommendations to restrain from light exposure during the night (employee 3) experience significantly less social jet-lag. Note, however, that being exposed to insufficient light levels during the day can also result in having spontaneous wake times after the alarm, even in the absence of evening light. Besides circadian adherence, the location in the office also affects sleep. Employees that sit close to the window (employee 1) and are thus exposed to higher daytime light intensities experience relatively less social jet-lag (employee 1 compared to employee 2). Differences in chronotype, reflected here by a different intrinsic circadian period, lead to differences in social jet-lag. People with longer circadian periods, the so-called evening types (employee 4), are more susceptible to the effects of night light compared to people with shorter circadian periods (employee 1), despite the fact that they are exposed to the same lighting conditions. One has to note, however, that the intrinsic circadian period is not the only parameter that affects chronotype. Inter-individual differences in chronotype may also be attributed to homeostatic sleep drive (i.e., the accumulation or dissipation of sleep pressure), as well as other circadian parameters, such as sensitivity to light. We use these results as an inspiration point and aim to counter-balance the effects of evening night exposure by providing personalized light (at the right levels and right times) that is able to introduce shifts in the circadian process. 

## 3. Mathematical Formulation of the Lighting System

We consider a dimmable LED lighting system denoted as $w=\left[w_{1},\;\ldots ,w_{N}\;\right]$, where $w_{i}$ is the dimming level of the i-th office hour ($0\leq w_{i}\leq 1$). In this paper, the vectors are represented in bold. For the *i*-th office hour, $w_{i}=0$ indicates that the lighting system is turned off, while $w_{i}=1$ indicates that the LED emits light at its maximum power. The lighting levels during office hours are given as:

(14) $$I=I_{max}w$$

where $I_{max}$ corresponds to the illuminance level [lx] when the lighting system is fully turned on. 

### 3.1. Optimization Target

Given the system dynamics (1)–(13), the optimization goal is to minimize the experienced (by the user) social jet-lag, quantified as the timing difference between the unconstrained wake-up times ($t_{w\_sp}$) and the enforced (by social constraints) wake-up timing ($t_{enf}$), by controlling the light according to dimming scenario w. Ideally,$\;t_{w\_sp}=t_{enf}$, yet this is not necessarily possible since the optimization is constrained by user lighting preferences and energy consumption, as energy efficiency is an essential requirement of modern lighting systems. 

### 3.2. Energy Consumption

The energy consumption of LED has a linear relationship with its dimming level [25]. Thus, the energy consumption of the lighting system can be formulated as a normalized function of the dimming vector as:

(15) $$\varepsilon \left(w\right)=\frac{pw+E_{0}}{p+E_{0}},$$

where p is the LED undimmed power consumption and$\;E_{0}$ is the standby energy consumed by the LED, which is independent of the dimming level. 

### 3.3. User Preference

**Figure 3:**
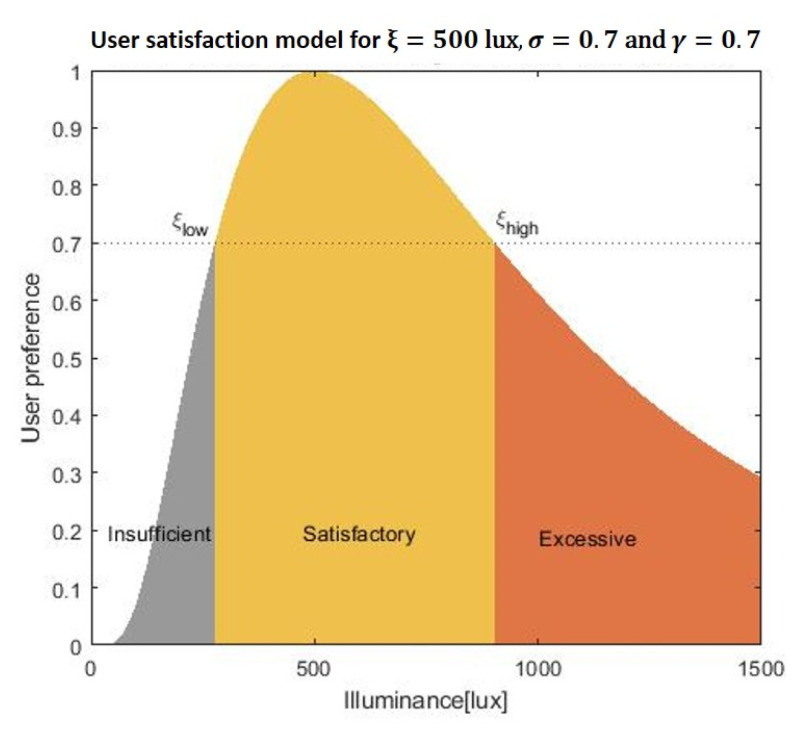
Log-normal user satisfaction model with the preferred illuminance at 500 lux. An illuminance level *x* is regarded as “Satisfactory” if and only if U(x)≥γ. Here, γ=0.7.

Lighting is supposed to deliver a level of comfort and is an essential factor in productivity and safety in professional settings. That is, circadian lighting cannot be seen as an isolation object, but must be considered in relation to the acute visual effects of light. Although the structure of the visual system is the same for everyone, there are still subtle differences among individuals. For example, people have different sensitivities to light or may have different refractive errors such as myopia, hyperopia, astigmatism, and presbyopia. As a result, the preferred lighting levels vary widely among individuals, depending on several factors such as gender, age, and culture [26,27]. The biggest challenge here is that we need a quantitative model of user satisfaction for light. However, transferring the subjective feelings of humans to quantitative measures is a non-trivial task. While most research on human light perception is mainly from a psychological or biological perspective, there are several attempts that opt for the mathematical interpretation of user preference appraisals. In this work, we assume a known quantitative user satisfaction model for light, following the approach presented in [28]. According to Fechner’s law, the eye senses brightness approximately logarithmically over a moderate range. Thus, we model user satisfaction as a function of the average illuminance of the task area as a log-normal shaped function. Over a practical range of illuminance, the appropriateness of the average task area illuminance can be modeled as:

(16) $$U\left(x\right)=\exp\left(-\frac{{\left(\ln x-\ln\xi \right)}^{2}}{2\sigma^{2}}\right),$$

where ξ corresponds to the most preferred illuminance of the user. Here, the parameter sigma (σ) describes the tolerance to illuminance of each individual; some people are only satisfied with a small range of illuminance, while others are more tolerant. As can be seen in Figure 3, the corresponding user satisfaction curve is smooth around the preferred lighting level and allows us to naturally interpret the user satisfaction as the acceptance probability of the lighting system. It is reasonable to assume that individual user is satisfied with an interval of illuminance $\xi_{low}\leq \xi \leq \xi_{high}$, where $\xi_{low}$ and $\xi_{high}$ are the lowest and highest illuminance values (in lux) that satisfy the user. In order to determine $\xi_{low}$ and $\xi_{high}$, we model the existence of a satisfaction threshold γ. An illuminance x is regarded as “Satisfactory” if and only if U(x)≥γ. Similarly, we can derive the probability that x is perceived as “Insufficient” and “Excessive” as:
SatisfactoryAll *x* for which U(x)≥γ,InsufficientU(x)<γ and x< ξ,ExcessiveU(x)<γ and x>ξ.


The preference learning framework used to train the user satisfaction model from the previously observed preference information is out of the scope of this work. Examples include [29,30]. 

### 3.4. Optimization Algorithm

The goal is to optimize the dimming vector w—i.e., the light levels during office hours—such that we minimize the social jet-lag experienced by the user while both conforming with user lighting preferences and staying below a predetermined energy budget. Thus, the lighting control problem may be formulated as a constrained optimization problem as:

(17) $$w^{*}=argminf\left(I=I_{max}w\right)=\|t_{w\_sp}-t_{enf}\|.$$

This is subject to:

0≤w≤1,
$\xi_{low}\leq I\leq \xi_{high}$,
$\varepsilon \left(w\right)\leq E_{L}$,

where $E_{L}$ is the given energy consumption (energy budget). 

Here, $f\left(I\right)=t_{w\_sp}-t_{enf}$ is the objective function. The enforced wake-up time $t_{enf}$ is the time that the user sets his/her alarm, while the spontaneous wake-up time $t_{w\_sp}$ is derived by the mathematical model described by the set of Equations (1)–(12), using $I=I_{max}w$ as the input in Equation (4). The first constraint is a physical requirement that the dimming level of every LED should be between 0 and 1. The second constraint is used to provide light levels that are considered “Satisfactory” by the user (U(x)≥γ), while the third constraint restricts the energy consumption below the maximum energy budget. 

However, the relationship describing the system dynamics (1)–(13) is highly non-linear, and the impact of w on $t_{w\_sp}$ is not direct, but requires the evaluation of the process for several hours after time *i*. As a result, the objective function is no longer guaranteed to be convex. Optimizing the dimming vector is equivalent to finding the global optimum of a non-convex function. As a result, a closed-form expression for w on $w^{*}$ does not exist, and more advanced optimization approaches are required. To solve this constrained non-convex optimization problem, we suggest a novel iterative optimization method that exploits the fact that light is more biologically effective at certain times of the day than at others by allocating more energy (increasing the light level) to the time slots that are able to introduce larger shifts in the spontaneous wake-up times. The algorithm works by computing the “sensitivity” function:

(18) $$\lambda_{i}=\frac{dt_{w\_sp}}{dw_{i}},$$

which relates changes in the input vector w to changes in the optimization function. 

At each step, the algorithm takes the previous dimming vector, and, using the sensitivity function, computes a set of small changes which, when added to the dimming vector, will decrease the optimization function while keeping the constraints satisfied. We use an iterative scheme that operates by increasing the light level step by step in appropriately chosen time intervals. The underlying principle is to take the interval where the derivative is the largest—i.e., the interval of additional light that has the highest impact. Formally, in the *k*-th iteration step, we wish to allocate an incremental amount of energy to the time slot $i_{k}$, that then maximizes the sensitivity function—i.e., we chose:

(19) $$i_{k}=\begin{array}{c} \mathrm{argmax}\\ i \end{array}\lambda_{i}.$$

Starting from an initial state, we iteratively add a small increase to the current state (small increase in light level) to the *i*-th dimension of w (*i*-th time slot), according to:

(20) $$\overset{´}{w}=w+ve_{i},$$

where $e_{i}=\left[0,\;0,\;\ldots ,\;1,\;0,\;\ldots \right]$ is the unit vector of the *i*-th dimension and v is a step-size variable that determines the amplitude of the increase. In every iteration, the objective function $f\left(I=I_{max}w\right)$ is evaluated using (1)–(13) until the next forecasted wake up event. We accept as a new state, the dimming vector $\overset{´}{w}$ that has the lowest objective function according to (18). The process is repeated until convergence or when the power constraint is satisfied. The stopping criterion is introduced to verify that w has reached or approximately reached the fixed point and that the objective function cannot be further improved. The pseudocode of the optimization algorithm is shown as the ALPHA-CO (**A**daptive **L**ight **P**ersonalization for **H**um**A**n **C**ircadian **O**ptimization) (Algorithm 1).
**Algorithm 1:** ALPHA-CO.Initialization: Set k=0. Set the initial control input to the minimum satisfactory lighting levels $w_{0}=\xi_{low}$
**while**$\;\varepsilon \left(w\right)\leq E_{L}$ and $\left|f{\left(I\right)}_{k}-f{\left(I\right)}_{k-1}\right|\leq \varepsilon$ do
**for**i=1 to *N* do
$\overset{´}{w}⇐GenerateDimmingVector\left(w,i\right)$ according to (19)
$\overset{´}{I}=I_{max}\overset{´}{w}$Evaluate (1)–(13) until $t=t_{w\_sp}$ and determine the sensitivity function $\lambda_{i}$
**end for**$w⇐\overset{´}{w}$ that maximizes λ
$I_{k}=I_{max}w$Calculate ε(w)
Increment k by 1
**end while**Output $w^{*}$


## 4. Results 

**Figure 4:**
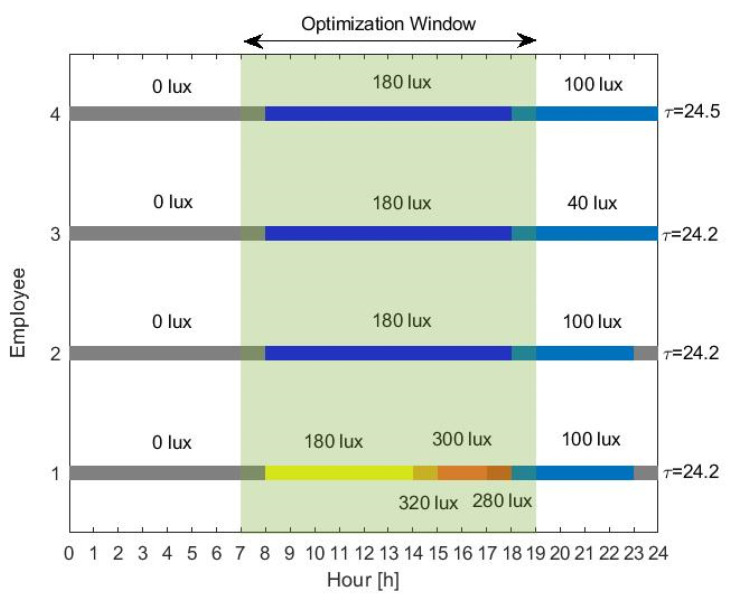
Lighting profile for every employee (1–4) input to the circadian pacemaker. We considered that light can be tuned (optimized) during the office hours (shaded area).

This work describes an adaptive office lighting system; thus, we assume that light can be tuned only during the office hours (between 08:00 h and 19:00 h). For the simulations, the lighting profile for each employee is presented in Figure 4, representing what we consider as typical light levels in modern societies. Employees 1 and 2 are exposed to relatively bright light during the late evening and night. However, employee 1 receives more light during the day, representing a user that sits closer to a window or is located at a brighter office. The third employee receives low light levels during the day but restrains from light exposure during the night by, for example, turning off or dimming the lights in the living room. We consider that such users are still engaged in some kind of activity during the night—for example, watching TV or mobile phone usage. A fourth employee is simulated who is exposed to exactly the same lighting as the second, but has a different chronotype. Here, the different chronotype is characterized by a longer circadian period τ=24.5 h, slightly longer than the average τ=24.2 h, corresponding to what we would typically call an evening person. All the employees are required to wake up at a fixed alarm time (07:30 h). For the rest of the simulations, the model parameters were set to their average values, as listed in Table 1, unless otherwise stated. All the users have the same user preference curve, with ξ=500 lux, σ=0.7, and γ=0.7. Note that, in practice, given user-specific information—such as age or chronotype, parameters $\mu_{H}$ and $v_{vc}$ (age-related), and circadian period τ (correlated to chronotype)—may be tuned accordingly to match the particular user. Additionally, self-learning methods can be used to derive a personal light preference curve for every user. Light is the input to the mathematical model through Equation (4), and spontaneous sleep and wake-up times are determined by the mathematical model and compared to the enforced (by alarm) wake-up time. The suggested optimization algorithm is implemented on MATLAB, assuming 10 days of training under the same dimming vector w. 

**Figure 5:**
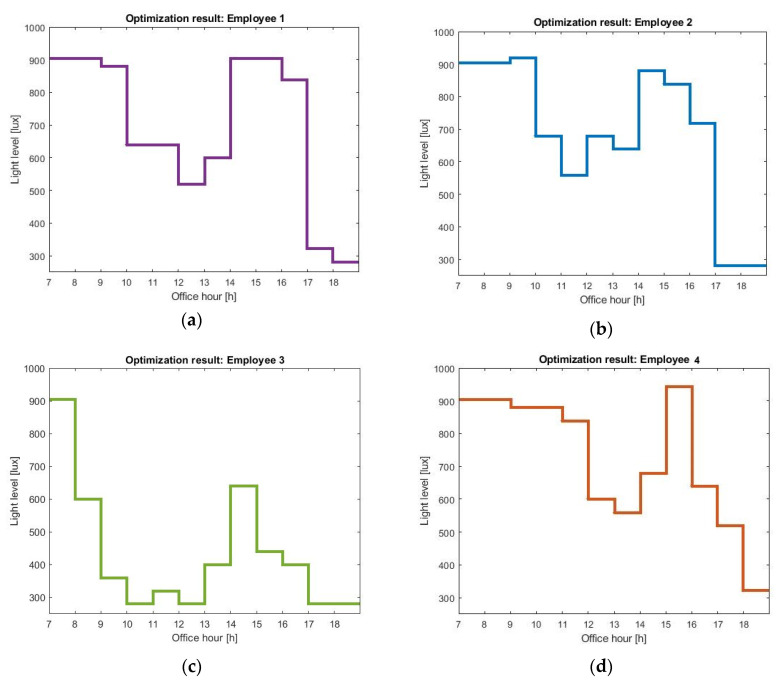
Optimal lighting schedules during office hours to shift the employees wake-up time. (**a**) Employee 1 receives light during the early morning (phase advancing region), followed by a dip in the light levels during the mid-day (dead-zone in the pacemaker), followed by an increase during the early evening (amplitude attenuation region); (**b**) employee 2 also receives more light in the phase advancing region, followed by a dip during the mid-day, followed by an increase during the early evening (amplitude attenuation region); (**c**) employee 3 receives light during the phase advancing region (early morning), followed by a larger dip in the light levels during mid-day, followed by a moderate increase during the early evening; (**d**) employee 4 light exposure is shifted by approximately an hour compared to employee 2.

Each optimal schedule in Figure 5 gives the pattern of light during office hours, which will shift (in our case, advance) the employees wake-up times to earlier in the day. The optimal profiles are different for every user, suggesting that history of light exposure, user habits, and preferences are key to personalized lighting systems.

All the profiles show increased light levels during the early morning (07:00 h to 9:00 h). That prediction is supported by quite a few studies that report daytime light exposure to be supportive for good sleep and wellbeing [31,32,33], and laboratory experiments that studied the response of the human pacemaker to light stimuli; light during the end of the biological night (early morning) results in phase advances, while light administered at the beginning of the biological night delays the clock [34,35]. Our optimal light schedules involve a dimming in the light exposure during the middle of the day. The authors in [36] showed that the solution to minimal entrainment time is bang-bang—i.e., optimal schedules consist of periods of either darkness or maximum light levels. Indeed, when the optimization is not subject to any constrains on personal preferences and available energy budget, the resulting schedules would involve having the brightest allowed light level at all time intervals during office hours, followed by an abrupt switch to darkness during the phase-delaying zone of the circadian pacemaker. However, our application of interest—namely, bio-adaptive office lighting—is not designed as a one-time intervention but as an office solution where the lighting levels are constantly adjusted to meet individual needs. As a result, the goal of the optimization is not minimizing the time to entrainment but achieving the maximum possible shift given the available resources. As a consequence, the resulting lighting schedules dim the lighting levels during the time intervals that are comparatively less biologically effective (e.g., the time interval that increasing the light levels does not lead to large phase shifts). One has to note that light during the mid-day is still able to introduce shifts in the circadian pacemaker; however, its effect is less compared to light during the early morning. Interestingly, we observe an increase in the light levels in the early afternoon. Taking a closer look at the limit cycle model in an attempt to explain the resulting light levels, we see that increasing the light in the early afternoon increases the amplitude of the van der Pol oscillator, as captured by Equations (1) and (2). Interestingly, we did see experimental studies [37,38] that investigate the effects of late evening exposure on physiology and sleepiness, specifically when this light exposure is preceded by early evening light. Yet, seeing also the experimental studies in [39] that showed that high-amplitude rhythms introduce larger phase-shifting responses, we hesitate to claim that our model explains their findings via amplitude shifts, but recommend further study. The timing of afternoon light exposure depends on the user chronotype. Employee 4, having an intrinsic circadian period larger than the 24 h cycle, has a circadian clock that oscillates at a slower pace than the wall clock. As a result, the dip in the light levels is slightly shifted and the maximum light level is administered at 15:00 h in a 1 h shift, compared to the maximum light level for employee 2. For a user that does not show extended light exposure during the night (employee 3), a decrease in sensitivity is not of value any more, and thus the optimization suggests relatively reduced afternoon light levels.

**Figure 6:**
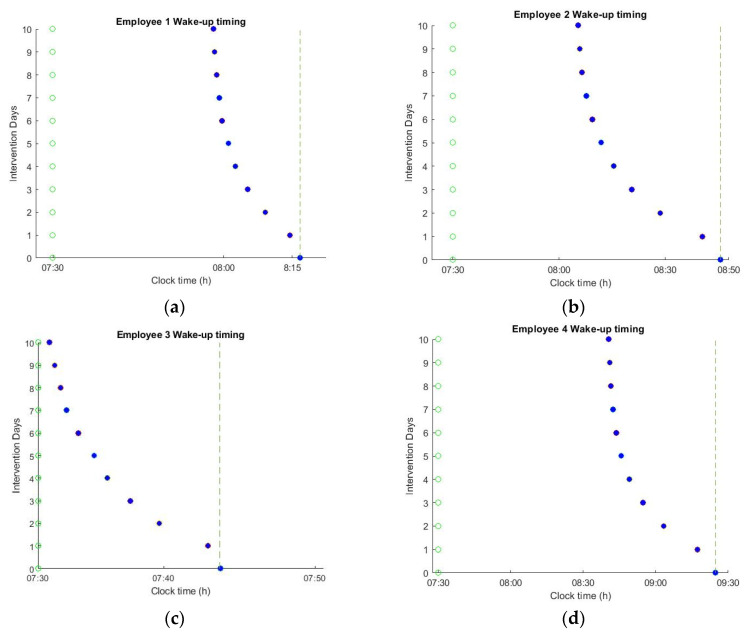
Predicted shift in wake-up times as a result of applying the optimal lighting schedules. (**a**) Employee 1 wake-up time is shifted by ~15 min; (**b**) employee 2 wake-up time is shifted by ~38 min; (**c**) employee 3 wake-up time is shifted by ~12 min; (**d**) employee 4 wake-up time is shifted by ~45 min.

**Figure 7:**
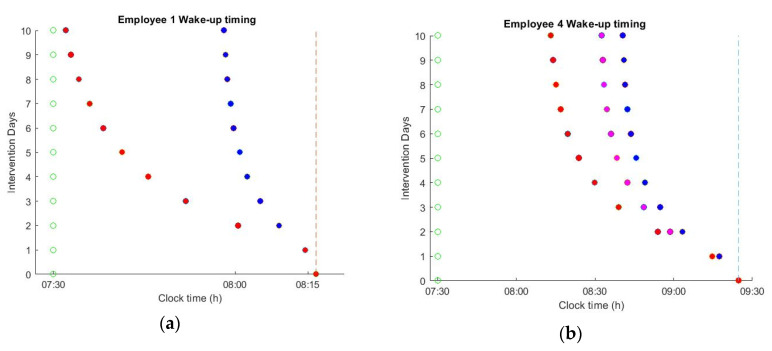
Predicted shift in wake-up times as a result of applying the updated lighting schedules. (**a**) Employee 1 wake-up time is shifted by ~40 min (red points) when reducing night light consumption by 30%; (**b**) employee 4 wake-up time is shifted by ~ 51 min when morning light is increased to excessive levels (magenta points), and further shifted by ~ 73 min (red points) when night light consumption is reduced by 30%.

The impact of applying the optimal schedules on spontaneous wake up timing is shown in Figure 6. The resulting energy consumption of the lighting system is reported in Table 2 as percentage over baseline illumination. During the 10 days of intervention, the natural sleep and wake-up times were gradually shifted to an earlier time point. However, the effects of changing the office lighting schedule depend on the intrinsic circadian period, light history, and user habits. For example, employee 3 has a routine that is characterized by low levels of light exposure during the night. For such users, the optimal lighting schedule was able to shift the wake-up times by ~10 min, aligning the natural wake-up time with the alarm in only 10 days of lighting intervention. However, for the employees that have a rather extended night light exposure, such a drastic intervention is not possible. For example, for employees 2 and 4, the optimal schedules were able to shift the wake-up times by ~38 and ~45 min, respectively. However, despite this extreme shift, it was still not possible to achieve the targeted alarm time. For such employees, dimming the light levels during the night or the acceptance of some instantaneous discomfort during office hours is essential to safeguard a longer-term positive effect on circadian well-being. Figure 7b shows the resulting phase-shift for employee 4 at a new light schedule that may no longer be considered satisfactory by the user at all times. In particular, light exposure is “excessive” during the early morning and afternoon, which, in combination with a 30% reduction in the evening light exposure, results in more than 60 min of phase shift. Employees that already received sufficient lighting during the day are the ones who are helped the least by the change in the office lighting (Figure 6a). Since the human dose response curve to circadian phase resetting is highly non-linear, the resulting phase-shift saturates after reaching a sufficient light level ~550 lux at eye level (which corresponds to a 1100 lux desk illuminance) [40]. This saturation point is effectively predicted by the mathematical model. Thus, after reaching a sufficient light level the benefit of further increasing the light level is negligible compared to the annoyance it causes to the user. For such users, reducing the light levels during the night is the only way to achieve the desired phase shift (Figure 7a). The reduction of evening light consumption is relatively more effective, since the relationship between day-time and evening light exposure is highly nonlinear, with large increases in day-time light intensity needed to offset small increases in evening light intensity [23]. 

**Figure 8:**
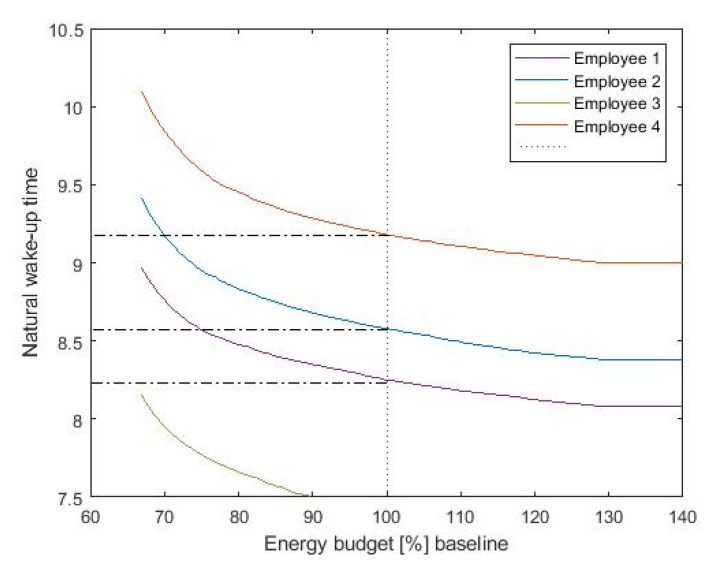
Resulting wake-up times as a function of the available energy budget. At 100% of the baseline energy budget, the optimization algorithm is able to shift the wake-up times of employee 2 by 12 min and employee 4 by 15 min, while employee 3 is able to reach the targeted alarm time at 90% of the baseline energy.

The resulting shift in the wake up times highly depends on the available energy budget. Figure 8 shows the achieved wake-up time as a function of the available budget, presented here as percentage over baseline illumination. We consider as typical baseline illumination a fixed lighting level of 500 lux during office hours. The available budget determines the shift we are able to introduce with lighting. As more budget is available, we can further increase the light levels, introducing larger clock shifts. Note that even at baseline energy consumption (100%), the optimization algorithm is able to provide sufficient circadian stimulus to shift the wake-up time while preventing excessive energy use by strategically applying light at the most biologically effective times and reducing energy consumption at the times when light input is the least effective.

## 5. Conclusions

Light regulates human physiology and behavior, perhaps most notably the sleep-wake cycle, by directly stimulating the internal timing mechanisms of the brain. Since humans spend over 90% of their time indoors, the light in the built environment should be designed to affect circadian rhythms. This paper introduces a human centric intelligent office lighting control system that aims to support human circadian biology and health. The modern lifestyle is characterized by a mismatch between circadian and societal (e.g., work, school) clocks, a condition known as social jet-lag, which can have severe impacts in our health and well-being. We suggest to use office light to reduce social jet-lag by efficiently shifting the employee’s circadian clock. The lighting control system is formulated as a constrained optimization problem, where minimizing social jet-lag, quantified as the difference between spontaneous wake-up times and user alarm, is used as a performance metric. This optimization problem is highly non-linear. To address this, we formulated and analyzed a novel ALPHA-CO adaptive optimization strategy to provide a solution.

While a further validation of our proposed algorithm against human data could not yet be performed, our initial results suggest that appropriately timed office lighting is able to introduce shifts to the internal circadian timing and align employees natural wake-up and alarm times, where a comparison with previously reported experimental work consistently agreed with our findings. The outcomes of the mathematical model give quantified results that can be used in lighting control and can be generalized using human-specific inputs. The trends agree well with qualitative, averaged findings in experimental user tests of the reported earlier literature. In fact, our model-based light recipes can cope with arbitrary light sequences and can allow user interventions, while the experimental test reports thus far were all fundamentally limited to specific scenarios. In particular, based on our optimization results, we are able to identify two modes of entrainment—namely, through increasing the pace of the limit cycle oscillator or through amplitude attenuation. Light introduced in the early morning is able to shift the wake-up time by means of accelerating the speed of the oscillator. Alternatively, strategically applying light at regions that attenuate the oscillation amplitude is able to mitigate the delaying effects of evening light exposure. Towards this end, individual light consumption is a key, as the level and timing of light intervention highly depend on the previous light history and individual habits. In particular, individual differences in late-evening light exposure require differentiation in the late afternoon exposures.

## Figures and Tables

**Table 1:** Model parameter values.

Circadian Model Parameter	Value	Sleep Model Parameter	Value	Sleep Model Parameter	Value
μ	0.13	$\mu_{H}$	4 nMs	$v_{vm}$	2.1 mVs
k	0.55	$v_{vc}$	2.9 mV	$v_{mv}$	1.8 mVs
$a_{0}$	0.1	χ	45 h	$D_{m}$	1.3 mV
β	0.007	$v_{vh}$	$1{\;\mathrm{mVnM}}^{-1}{}^{}\;$	$v_{vm}$	2.1 mVs
G	37	$D_{0}$	10.2 mV	$v_{mv}$	1.8 mVs
τ	24.2 h	$Q_{th}$	$1{\;s}^{-1}$	$D_{m}$	1.3 mV
b	0.4	$\tau_{v,m}$	10 s	$v_{vm}$	2.1 mVs

**Table 2:** Energy consumption.

Employee	Energy Consumption [%] Baseline Illumination
1	**130%**
2	**129%**
3	**90%**
4	**133%**
4 (lighting schedule with excessive light levels during the early morning)	**142%**
